# Smart Accessibility: Design Process of Integrated Geospatial Data Models to Present User-Customized Universal Design Information

**DOI:** 10.3389/fpsyg.2019.02951

**Published:** 2020-01-14

**Authors:** Sooyeon Rosie Han, Seunghyun Yoon, Suyeon Cho

**Affiliations:** ^1^Urban Computing Research Lab, Konnektus, Seoul, South Korea; ^2^IT Convergence and Security Lab, Pluxity Co., Ltd., Seoul, South Korea

**Keywords:** accessibility, barrier-free, geospatial, information model, universal design, user-customized, vulnerable groups, environmental behavior

## Abstract

Environmental accessibility information measured by universal design guidelines does not exist in a form that can be effectively implemented as a geospatial database. Thus, this study explored the design process of a smart accessibility data model that integrates geospatial data with environmental accessibility information in a mixed indoor/outdoor environment. First, a typology of accessibility information integrated with universally designed geospatial information was identified, and a field experiment was conducted to observe components of the built environment and environment-behavior interactions during travel in a sightseeing area of Seoul, South Korea. The analysis found that each user group, namely people with mobility impairments, visual impairments, or hearing impairments, had different barriers, and facilitators in the environment. Certain barriers for one group could work as facilitators for another, and vice versa; also, some components previously classified as facilitators failed to actually fulfill that function. Additionally, the user groups demonstrated different prioritization of spatial attributes. The findings of the field study were organized in the data model as priority information and weighted values according to user group. The smart accessibility data model developed in this study has implications for designing user-customized multimodal systems, such as wayfinding services and web-based maps, that are useful to everyone, regardless of their ability or age. Furthermore, it increases the users’ decision-making power to plan a trip and can exert invisible pressure inducing physical improvements in the areas that lack accessibility, through visually displaying accurate information on the physical environment.

## Introduction

Vulnerable groups are more strongly affected by environmental factors that determine an individual’s degree of independent life and equitable rights to social participation (Iwarsson and Ståhl, 2003). Various attempts have been made all over the world to improve environmental accessibility, from developing instruments such as guidelines and checklists to measuring actual environmental conditions, at the local, federal, and national levels, and in collaboration with the private sector. However, since these instruments are not developed with no consideration for the design of a wayfinding system, the information collected is not in a form that can be effectively implemented as a geospatial database, which plays a key role in developing service platforms. Existing geospatial services, such as web-based maps and navigation applications, are thus intended primarily for people without disabilities, as they are based on geometric location data only, which cannot confirm the access, mobility, and safety of vulnerable groups. The few studies dealing with environmental accessibility in geographic information systems (GISs) concentrate only on people with visual impairments (Marsh et al., 2000; Ran et al., 2004; Farcy et al., 2006; Hunaiti et al., 2006) and people who use wheelchairs (Hunaiti et al., 2006; Karimi et al., 2014). They do not adequately provide user-customized information due to the limited source of data relying on governmental standards, focus mainly on outdoor data (e.g., pedestrian accessibility) although the environment is intimately connected by outdoor spaces and indoor spaces, and omit the process by which information should be stored mapped, and managed. Therefore, this study defines the concept of geospatial information for all, identifies typology of environmental accessibility, and finally presents a smart accessibility data model that integrates geospatial data with user-customized universal design information in a mixed indoor/outdoor environment, based on an empirical understanding of complex environment-behavior interactions.

## Research Methods

**FIGURE 1:**
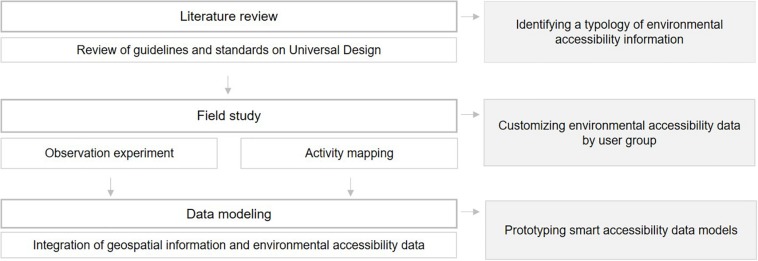
Overview and flow of research.

Figure 1 shows a general overview of the current study’s research method, outlining the primary steps required to design the smart accessibility data model.

Ten global guidelines or relevant research papers were reviewed to define the concept of universally designed geospatial information and to identify a typology of universally designed geospatial information, listing both geospatial data and attribute data. Universal design guidelines have been applied at all different scales, from electronic products to transportation systems. This study focused on environmental accessibility in the built environment at the local, federal, and national levels, in collaboration with the private sector, to obtain diverse environmental components.

To fill a gap in knowledge about environmental accessibility information drew in the previous stage and to examine the interaction process between components of the built environment and users with special needs, an observation experiment was conducted in a sightseeing area of Seoul. Significant differences according to user groups were found, addressed in the smart accessibility data model by priority information and values weighted by user group. This study took a microscopic approach to identify individuals’ purpose-driven mobility process and behavioral characteristics, directly observing and monitoring the reaction and behavior of a subject in particular locations. Observation is an effective method for vividly describing and analyzing subjects’ behavior or attitude and has been considered an effective data collection method when the nature of a phenomenon would be difficult to identify through questionnaires alone (Whyte, 2001; John, 2006; Gehl and Svarre, 2013). Behavior-centered observation focuses on the actions and reactions of the observed subject in a physical environment, whereas environment-centered observation focuses on changes in the physical environment based on the detailed evidence that appears there. During observations, it is important to carefully analyze changes in activity caused by the environment, as well as changes in the physical environment from the observer’s perspective. However, because it is difficult to control the intervention of unwanted external variables in a natural environment at the discretion of the observer, and because thorough observation of the selected samples over a long period of time is required, it is difficult to quantify and generalize the results of observations. This study thus focused on exploring in detail and qualitatively analyzing the development of a particular situation, which cannot be generalized, based on the physical characteristics of the subjects. Furthermore, to grasp causes of specific behaviors, unstructured interviews were conducted right after the experiment. To verify validity through detailed observation, more than two observers were placed on site for each type of subject.

The final step of the study was data modeling. Unified Modeling Language (UML) class diagrams were adopted to formalize this study’s smart accessibility data model. UML is a standard visual modeling language that has been used for the analysis, design, and implementation of software-based systems (Borges et al., 2001; Lisboa-Filho et al., 2010). The class diagram is the main building block of object-oriented modeling in UML, containing object classes, their attributes, and the relationships among objects. In a few significant studies, researchers have integrated information about environmental accessibility with a data model. However, the studies were limited in that the information pertained only to particular groups, such as people with visual impairments or people using wheelchairs due to mobility impairments (Chen et al., 2015; Kasemsuppakorn et al., 2015); in that they incorporated only certain types of environmental information, such as outdoor pedestrian accessibility (Yairi and Seiji, 2007; Laakso et al., 2013); and in that they presented the information model at a conceptual level only, instead of implementing actual field-based information. This study designed a smart accessibility data model intended for all people based on universally designed geospatial information in a mixed indoor/outdoor environment.

## Toward “Geospatial Information for All”

### The Concept of Universal Design in Geospatial Information

Universal design was first defined as “simply a way of designing a building or facility at little or no extra cost so it is both attractive and functional for all people, disabled or not” (Mace, 1985). Since then, the concept has been more broadly described by the European Institute for Design and Disability (EIDD) in terms of the “Design for All” philosophy, which aims to realize accessibility, convenience to all members of society, and responsiveness to evolving diversity in the built environment, everyday objects, services, culture, and information (EIDD, 2004). Similarly, the Health and Places Initiative (HAPI) presents seven principles of universal design: equitable use, flexibility in use, simple and intuitive use, perceptible information, tolerance for error, low physical effort, and size and space for approach and use (HAPI, 2014). Other related concepts include barrier-free and inclusive design, which share a focus in designing everything to meet the needs of all, regardless of ability or age.

**FIGURE 2:**
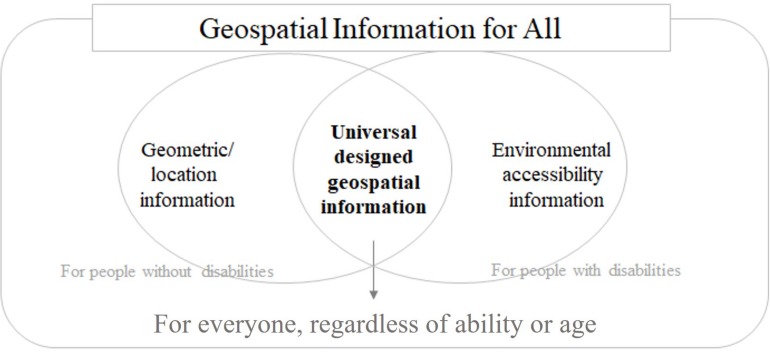
Conceptual diagram of “geospatial information for all.”

This study focused on information describing built environment in universal design approach, pursuing the goal of “geospatial information for all” (Figure 2). Geospatial information is data referenced to a place (i.e., a set of geographic coordinates) that can be gathered, manipulated, and displayed in a GIS (Folger, 2010). The information includes both geometric location data and attribute data, or descriptive information about the properties of events, features, or entities associated with a place. Existing geospatial information does not incorporate environmental accessibility information as attribute data, which leaves vulnerable groups unsure of their access, mobility, and safety. This is because environmental accessibility information, which is established and measured by the standards and guidelines of universal design, is not collected in a form that can be effectively implemented as geospatial attribute data. To provide geospatial information for all, information about environmental accessibility must be included, and further, geospatial information should be provided in the form of the “smart accessibility data model” described in section “Smart Accessibility Data Model.”

### Identifying a Typology of Environmental Accessibility Information

Several guidelines and standards for environmental accessibility have been established to compile requirements and measure environmental conditions at the local, federal, and national levels, in collaboration with the private sector (Table 1). Each guideline is categorized based on its place of application, such as streets or buildings, and each category includes features designed for people with visual impairments or wheelchair users with mobility impairments. The World Tourism Organization (UNWTO) provides an accessibility guideline for tourist attractions worldwide with the goal of realizing “Tourism for All,” so that there may be no grievances regarding accessibility or mobility in use of tourism resources (World Tourism Organization, 2013; World Tourism Organization and Fundación ACS, 2015).

**TABLE 1:** Example guidelines and standards of universal design.

**Standard/Guidelines**	**Country/Organization**	**Application**	**Status**	**Last Updated**
City of Toronto accessibility design guidelines	Canada	Built environment	Adopted	2004
Design guide for wheelchair accessible housing	United Kingdom	Buildings	Adopted	2006
2007 Facility accessibility design standards	United Kingdom	Building	Adopted	2007
Recommendations on accesible tourism for all	UNWTO	Tourist sites	Adopted	2013
Manual on accessible tourism for all	UNWTO	Tourist sites	Adopted	2014
Mobility, universal design, health, and place [research brief]	United States	Built environment	Adopted	2015
Accessibility design guide: universal design principles for Australia’s aid program	Australia	Built environment	Adopted	2015
Seoul universal design guideline	South Korea	Sidewalks, streets, buildings	Adopted	2017
Seongbuk universal design guideline	South Korea	Sidewalks, streets	Adopted	2018

Based on examination of the guidelines and standards outlined in Table 1, types of spatial and attribute data that needed to be built as a future data model were classified into two lists, for outdoor and indoor environments (Table 2). Spatial data for both lists included components of the built environment, such as entrances and vertical access (i.e., stairs, elevators or lifts, and escalators). Each component includes attribute data containing information about accessibility. For example, an entrance consists of a door and a ramp. Doors include attribute data such as the type and width of the door, the direction of opening, the height of threshold, and the type of knob, whereas ramps include attribute data such as the width, gradient, and texture.

**TABLE 2:** Typology of accessibility in indoor and outdoor environments.

Outdoor	Sidewalk	Pedestrian path	Width of path; material of path; gradient of path; location of boundary stone; surface materials
		Curb	Location of curbstone; height of curbstone
		Level changes	Height of threshold; change in paving stone
		Bollard	Location of bollard; height of bollard
	
	Vertical access	Stairs	Tread; height of steps/riser; height of threshold; type of steps; step floor material; level section of handrail
		Elevator	Width of hall doors; location of buttons/controls
		Escalator	Width of escalator
		Lift	Control panel; weight limitation; arrival call system
	
	Entrance	Door	Type of door; width of door; direction of opening; height of threshold; type of knob; location of knob
		Ramp	Width of ramp; gradient of ramp; texture of ramp surface

Indoor	Entrance	Door	Type of door; width of door; direction of opening; height of threshold; type of knob; location of knob
		Ramp	Width of ramp; gradient of ramp; texture of ramp surface
	
	Hall	Aisles	Width of aisle; texture of aisle; location of handrail
	
	Restroom	Accessible restroom	Accessible toilet signage; side grab bar
		Door	Type of door; width of door; direction of opening; height of threshold; type of knob; location of knob
		Basins	Location of basins; type of basins; location of faucets
		Braille	Location of Braille sign
	
	Vertical access	Stairs	Tread; height of steps/riser; height of threshold; type of steps; step floor material; level section of handrail
		Elevator	Width of hall doors; location of buttons/controls
		Escalator	Width of escalator
		Lift	Control panel; weight limitation; arrival call system
	
	Connection	Hall	Width of aisle; texture of aisle; location of handrail
	space	Door	Type of door; width of door; direction of opening; height of threshold; type of knob; location of knob

## Interaction Between the Built Environment and Special User Groups

### Field Experiment

Because the dynamic and comprehensive nature of environment-behavior interactions is difficult to capture through literature review, a field study was conducted in Seoul. The purpose was to classify attribute data by user group to provide user-customized universal design information; this was done by observing the significant differences stemming from various interactions between the building components and the user groups with special needs.

**FIGURE 3:**
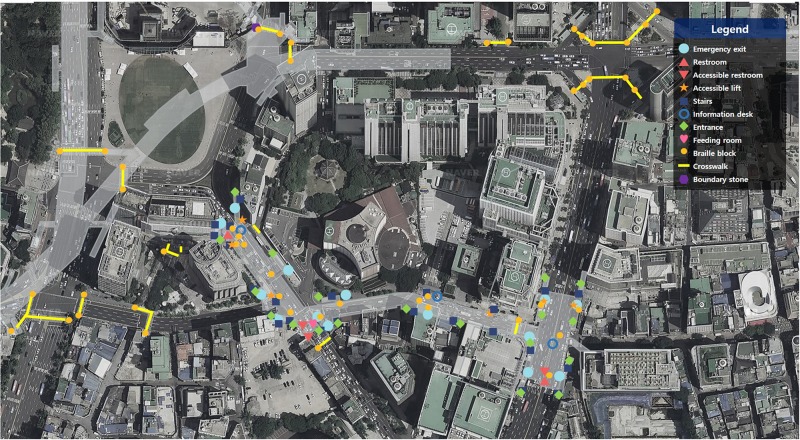
Area and environmental features of the field experiment.

The locations chosen for this study were situated in an underground shopping mall in Seoul. To focus on interactions during continuous travel from indoor to outdoor space, locations with many connecting spaces were selected, such as subway stations, department stores, the city hall, and public squares (Figure 3). The physical environment was studied to map all physical conditions or variables in the targeted space that could influence people’s actions, and this study was analyzed along with a floor plan analysis and the field experiment. Prior to the experiment, the floor plan and components of the space and their relationships were examined to investigate the environmental features from the perspective of universal design. The attribute information of the targeted locations was then outlined based on the lists for indoor and outdoor environments derived from analysis of existing guidelines.

The City Hall station was assigned as the starting point and Exit 18 of the underground shopping center was assigned as the arrival point. Participants were encouraged to move and explore freely between these two points, with the restrooms, nursing room, and shops assigned as required stops. For average pedestrians without any physical impairments, the total travel distance is 500 m; the travel time is approximately 15 min at a normal pace when the purpose of traveling is simply to move from the starting point to the arrival point. We speculated that there would be differences in methods of access and travel routes based on the characteristics specific to different user groups. Considering that the subway station and underground shopping center are a connected space for pedestrians, and to elicit a natural flow of movement, no time limitation was set for the study subjects to complete the field experiment. The average travel time spent on the observation experiment was approximately 44 min per person, ranging from 32 to 70 min. The experiment was conducted for 10 days, from September 17 to 28, 2018, during non-rush hours in the morning and afternoon to avoid heavy pedestrian traffic.

### Target Group

All people benefit from universal design, not only those with disabilities. However, this study’s experiment targeted people with special needs because the goal was to extract not only geometric location information, which is also accessible to people without disabilities, but also environmental accessibility information, which can change based on physical attributes or needs. To address the full variety of special needs and complete the list of attribute information present in domestic and international guidelines, which have thus far concentrated only on wheelchair users and people with visual impairments, user groups were roughly categorized into three groups: people with mobility impairments, people with visual impairments, and people with hearing impairments. People with mobility impairments included those who travel in wheelchairs and those who push baby strollers. Elderly people, who manifest complex physical attributes, were excluded from the experiment group, as their needs can be addressed by aggregating the attributes of each group. Attributes and assistive device features by subject type are presented in Table 3. To fully understand the characteristics of each user group, all subject types had physical attributes that were distinct from others in the same user group, and each type had more than two subjects. A total of 18 participants (eight males and 10 females; four wheelchair users; four people with babies in strollers; six people with vision impairments; and four people with hearing impairments) volunteered to take part in the experiment. The mean age of the participants was 35, ranging from 27 to 50 years. To verify validity through detailed observation, more than two observers were placed on site for each type of subject. Additionally, to exclude habitual traveling conditions that occur in familiar places, subjects were limited to those who had never been to the target area.

**TABLE 3:** Types and characteristics of study subjects.

**Subject Types**			**Attributes and Assistive Device Features**
People with mobility impairments	Wheelchair users	A-1	Uses a motorized wheelchair controlled by a head controller Searches for information on a tablet attached to the wheelchair, grabbing a stylus in the mouth due to immobility of both hands
		A-2	Uses a motorized wheelchair Operates a smartphone (mobility in both hands)
	People with stroller baby	B-1	Accompanied by an infant or baby under 10 months of age who cannot walk Use of a stroller is essential; needs diaper changing facilities; needs breastfeeding facilities
		B-2	Accompanied by a toddler under 3 years of age who is highly dependent on a stroller No need for diaper changing facilities, but need for children’s to
People with vision impairments		C-1	Partial blindness as an acquired disability; able to recognize objects by shape to a small degree Uses a smartphone with voice assistance
		C-2	Complete vision loss Uses a white cane; uses a smartphone with voice assistance
		C-3	Complete vision loss Accompanied by a guide dog; uses a smartphone with voice assistance
People with hearing impairments		D-1	Difficulty understanding written text due to the limited literacy Reliant on sign language
		D-2	Acquired hearing loss
			Understands written text and oral method; uses a smartphone

### Activity Mapping

**FIGURE 4:**
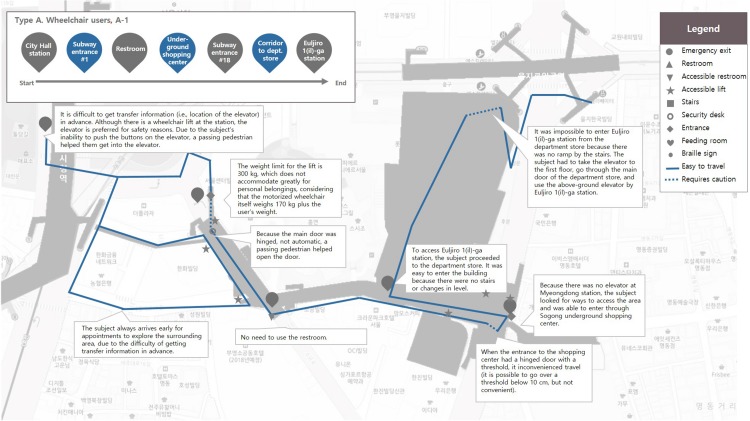
Sample activity mapping sheet in the group with mobility impairments.

A researcher conducted a one-to-one follow-up observation of a subject and recorded the observation on the prepared map. The results of follow-up interviews were recorded on the map. Mapping of observation points at a certain location is called activity mapping. The effectiveness of this research method is well-established and it is often employed in urban studies to understand the close relationship between user behavior and the physical elements of the environment (Appleyard, 1969; Gehl and Svarre, 2013). The observation and documentation in activity mapping focuses mainly on the physical access behaviors of the subject on the pathway from the starting point to the destination, such as gaze movements and directional changes at a stop. To ensure the collection of natural and realistic data, the researcher maintained a certain distance from the subject during the observation and documentation, to keep the subject from becoming conscious of the researcher. A sample map and observation documentation for one of the experiment subjects among total thirty-six cases is shown in Figure 4.

### Observation Results and Insights

The observation experiment showed significant differences in the needs of each user group and their interactions with the environmental elements. First, each group had a different focus: for people with a mobility impairment, physical accessibility was the greatest issue; for people with a visual impairment, orientation was the greatest issue; and for people with a hearing impairment, comprehensibility was the greatest issue.

Second, each user group had different environmental barriers and facilitators, and the same spatial information that functioned as a barrier to one group could function as a facilitator to another (Table 4). For example, whereas the sidewalk curb is a barrier to access for people with a mobility impairment, it is a facilitator for people with a visual impairment, as it helps to distinguish a sidewalk from the road. Similarly, overpasses are a barrier for people with mobility impairments but safer paths than crosswalks for people with visual impairments. Conversely, elevators functioned as a facilitator for people with mobility impairments, but were avoided by people with visual impairments due to difficulty in operation and evacuation. If a building had even a single flight of stairs, people with visual impairments preferred to use the stairs.

**TABLE 4:** Barriers and facilitators for each user group.

**User Group**	**Barriers**	**Facilitators**
People with mobility impairments	– Sidewalk width less than 90 cm.	– Sidewalk separate from street
	– Gradient over 8%	– Lowered curb at crosswalk
	– Uneven surface	– Elevators
	– Misplaced street trees	– Lowered main entrance threshold
	– Crosswalk with curbstone	– Ramp
	– Stairs, escalator	– Automatic door
	– Overpass	– Wheelchair-accessible restrooms
	– Threshold at the main entrance	– Feeding/nursing rooms
	– Revolving door	– Diaper changing table
	– Main entry width less than 90 cm	– Restroom with children’s toilet

People with visual impairments	– Mixed traffic street	– Sidewalk separate from street
	– Uneven surface	– Braille block
	– Crosswalk	– Accessible pedestrian signal (APS)
	– Lowered sidewalk curb	– Handrail on stairs
	– Revolving door	– Braille sign on handrails
		– Help button
		– Elevator braille buttons
		– Automatic door
		– Tactile and auditory information on signs and kiosk

People with hearing impairments	– Mixed traffic street	– Flashing emergency lights
	– Voice assistance	– Sign language services
		– Pictograms on signs

Third, the importance of attribute data differed by group, even with respect to the same spatial information (Table 5). For example, when information on the entrance was provided, door width was the most important detail for the group with mobility impairments, whereas opening type was the most important for the group with visual impairments. Similarly, in the case of restrooms, accessibility and location were the most important pieces of information for the group with mobility impairments, whereas gender separation and orientation was the most important for the group with visual impairments.

**TABLE 5:** Prioritization of attribute data by user group.

	**People with mobility impairments**	**People with vision impairments**	**People with hearing impairments**
Pedestrian Path	Gradient	With Braille block	
	Width		

Pedestrian Crossing	Crossing type	With acoustic signal	

Curb	Height	Missing	

Stairs	With ramp	With handrail	
	Ramp gradient	With Braille sign	
	Ramp width	Number of steps	

Elevator	Wheelchair-accessible	With help button	With strobe light
		With Braille sign button	

Corridor	Gradient	With Braille block	With strobe light
	Width		

Door	Width	Opening type	
	With threshold	Opening direction	
	Threshold height		

Room	Wheelchair-accessible		With strobe light

Restroom	Wheelchair-accessible	Distinction of gender	With strobe light
	With nursing room		
	With changing table		

Fourth, analysis of some observed points revealed misconceptions regarding some facilitators. For example, a Braille sign for gender marking was installed on the entrance of a restroom, but it was practically impossible for the group with visual impairments to locate, especially those with complete vision loss. Those with partial vision loss were able to locate the Braille sign, but the presence of the Braille was unnecessary because they were able to distinguish a men’s restroom from a women’s restroom based on the color and shape of the signs. Similarly, wheelchair lifts, which were initially classified as facilitators for people who travel in wheelchairs, were found to have limitations due to potential fall risk, the psychological burden of requesting someone’s help to operate the lift, and unwanted attention from other pedestrians when getting on the lift. Therefore, it is imperative to reflect the barriers and facilitators for each user group and the priority of attribute data when designing a data model.

## Smart Accessibility Data Model

### Data Network

**FIGURE 5:**
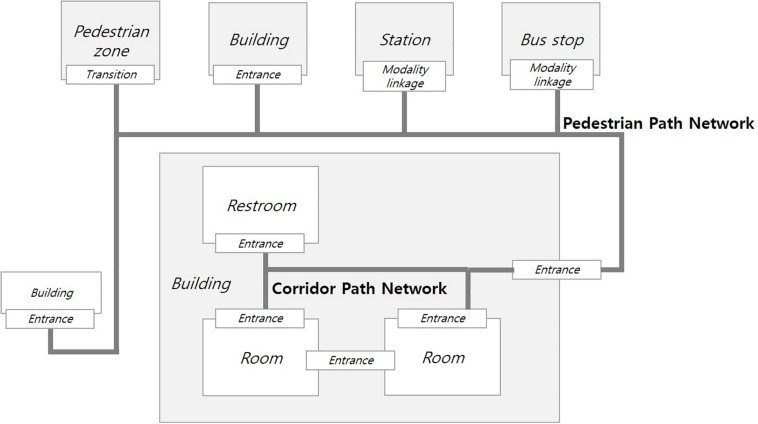
Topological network of the built environment.

The data model in this study was designed based on the topologically connected network linking the pedestrian path network of the outdoor environment to the corridor path network of the indoor environment (Figure 5). The pedestrian path network connects to pedestrian zones, such as parks and squares, by transition spaces; to buildings by entrances; and to train stations or bus stops by modality linkage. In other words, transition space connects open outdoor locations where it is difficult to define a specific entrance; entrances connect outdoor locations to indoor locations, or connect between indoor locations with a specific entrance area; and modality linkage connects places where the modality of movement changes, such as from walking to taking public transport. Similarly, the corridor path network in a building connects rooms or restrooms through entrances.

### Data Structure

**FIGURE 6:**
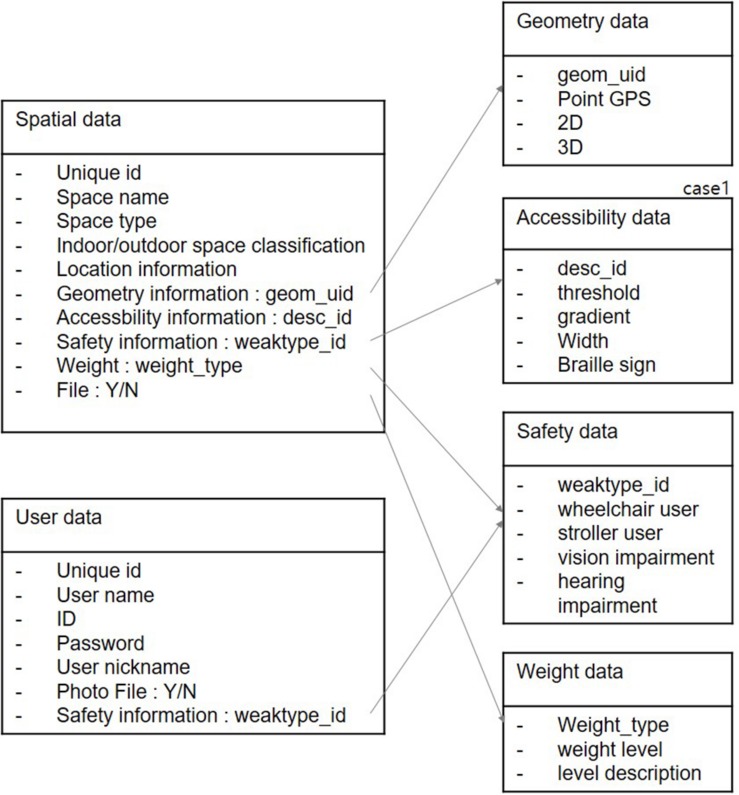
Universally designed geospatial data structure.

The data structure for providing user-customized universal design information is divided into universally designed geospatial data and user data (Figure 6). The universally designed geospatial data encompass not only location data, geometry data, and unique ID, which can identify the space by its purpose, but also accessibility, safety, and weighting information. Location data are divided into indoor data and outdoor data. When a subject is outdoors, geographic coordinates and altitude are displayed based on GPS information; when a subject is indoors, location information is provided using relative positioning based on the floor plan. Geometry data define GPS points that can be either two-dimensional (*x*, *y*) or three-dimensional (*x*, *y*, *z*). Environmental accessibility information includes attribute data extracted from the aforementioned typology of accessibility such as threshold height and braille sign. The data comprise safety information (referring to user data) and weighting information to evaluate the prioritization of attribute data by user type.

**FIGURE 7:**
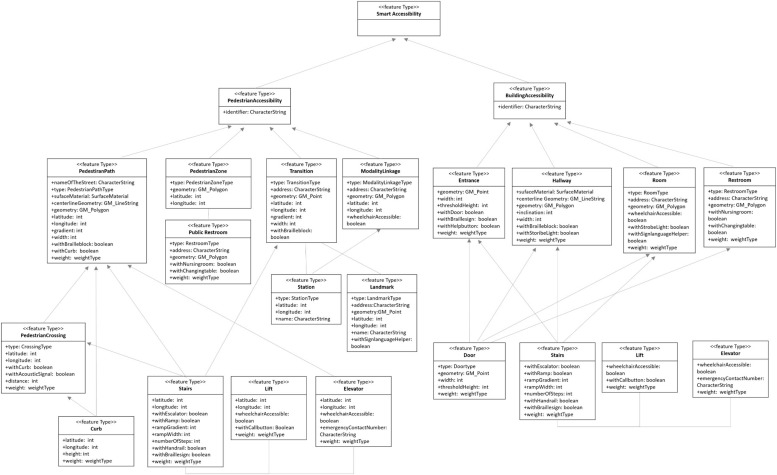
Smart accessibility data model.

Figure 7 shows the complete smart accessibility data model with inheritance relationships and associations. The model is divided into two top-level classes: Pedestrian Accessibility and Building Accessibility. The Pedestrian Accessibility class is inherited by the child classes Pedestrian Path, Pedestrian Zone, Transition, and Modality Linkage. In addition to typical attribute data, such as geometric type and latitude and longitude, the Pedestrian Path class includes attribute data for pedestrian path type, surface material, gradient, width, presence of a Braille block and curb, and weight type. Moreover, the Pedestrian Path class has relationship with Pedestrian Crossing, Curb, Stairs, Lift, and Elevator. The Pedestrian Crossing class has special attributes, such as presence of an acoustic signal, because this is important information for people with visual impairments. The Curb class can be associated with both the Pedestrian Path and Pedestrian Crossing classes due to level changes in the crosswalk area. The Stair class has attributes for the presence of a handrail and Braille sign and the number of steps, which is important information for people with visual impairments, and also ramp width and gradient, which is important for people with mobility impairments. The term “lift” is interchangeable with “elevator” in some countries; however, in the field experiment area of this study, the lift is an assistive device, distinct from an elevator, installed next to a staircase to aid the mobility of wheelchair users. The field experiment found that the wheelchair lift limited in functioning as a facilitator due to safety issues; therefore, it was not classified with a plus sign in weight type.

**FIGURE 8:**
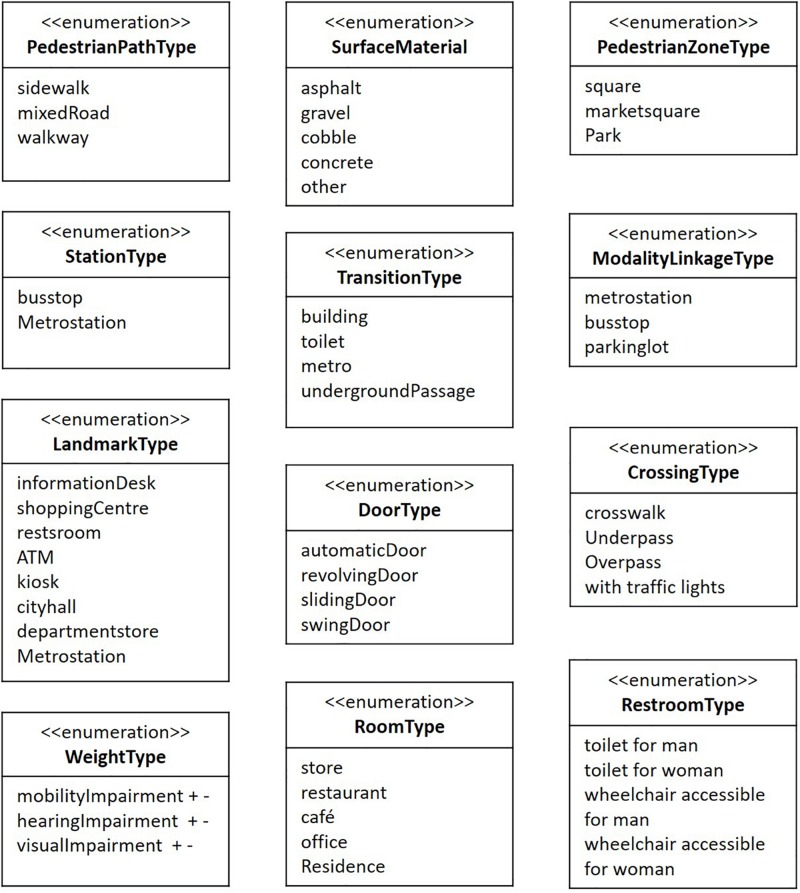
Enumeration of type information in attribute data.

**FIGURE 9:**
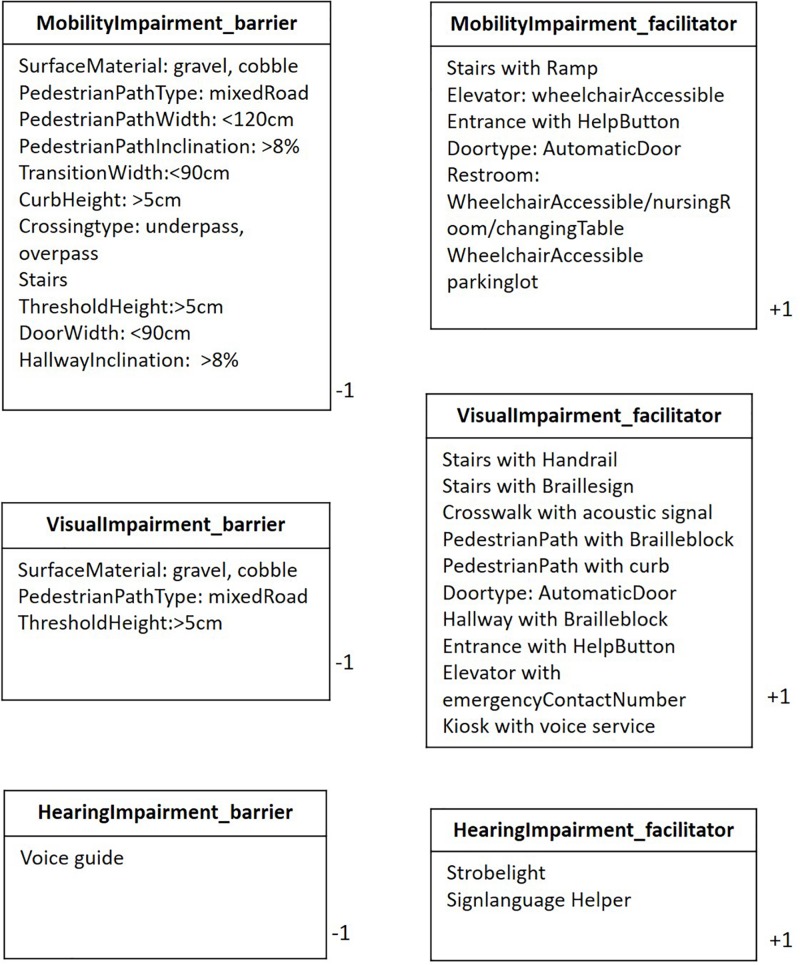
Weight type information for barriers and facilitators.

Type information in attribute data is defined as enumerations (Figure 8). For example, pedestrian path type is enumerated according to sidewalk, mixed road, and walkway, in that the risk level varies according to user type. Weight type includes user types such as visually impaired, mobility impaired, and hearing impaired, with plus and minus signs referring to information in the enumerations of barriers and facilitators (Figure 9). The weight value plays an important role in providing user-customized information because barriers and facilitators differ by user type. For example, certain barriers for people with a mobility impairment (e.g., a curb or pedestrian overpass) function as facilitators for people with a visual impairment; and even within the same spatial component, the priority of attribute data differs by user type, as captured from the field experiment.

## Discussion and Conclusion

This study was driven by three research aims. The first was to identify a typology of environmental accessibility information as geospatial attribute data by analyzing national universal and barrier-free design guidelines and standards. The second was to explore the different information needs and interaction methods of various user groups with the environment through an observation experiment. The analysis found that each user group, namely people with mobility impairments, visual impairments, or hearing impairments, had different barriers and facilitators in the environment. Certain barriers for one group could work as facilitators for another, and vice versa; also, some components previously classified as facilitators failed to actually fulfill that function. Additionally, the user groups demonstrated different prioritization of spatial attributes. The final research aim was to design a method to store and present information about environmental accessibility in a systematic way, resulting in smart accessibility data model that integrates geospatial data with user-customized universal design attribute data in a mixed indoor/outdoor environment. It should be noted that only static information was included as attribute information in the aggregated data of universal design. Dynamic information, such as construction status or floor condition based on the weather, was excluded.

To supplement and complete the existing universal design guidelines, which have accumulated data concentrating mainly on wheelchair users and people with visual impairments, this study’s experiment included participants pushing a baby stroller and people with a hearing impairment. In future, the range of participants can be extended to many other user types. Conversely, it is possible to develop an optimal approach by conducting more in-depth research on a particular user group.

The process of communication with users is a key issue, but faces limitations in displaying numerous pieces of attribute information using the conventional method. For example, people with visual impairments have limited access to visual maps; people with hearing impairments have a limited literacy and have limited access to voice assistance services. Thus, it is imperative to conduct a follow-up study on a multimodal system that provides information in the optimized way for users based on the smart accessibility data model presented in this study.

Providing information services using a geospatial database is as important as improving physical accessibility itself, which can take a relatively long time. It not only increases the users’ decision-making power but also visually displays accurate information on the physical environment, and can exert invisible pressure inducing physical improvements in the areas that lack accessibility.

## Data Availability Statement

All datasets generated for this study are included in the article/supplementary material.

## Ethics Statement

Ethical review and approval was not required for the study on human participants in accordance with the local legislation and institutional requirements. The patients/participants provided their written informed consent to participate in this study.

## Author Contributions

All authors contributed to the conception and design of the study, and manuscript revision, performed the content analysis and field study, and read and approved the submitted version of the manuscript. SH wrote the first draft of the manuscript. SY and SC wrote sections of the manuscript.

## Conflict of Interest

SH was employed by the company Konnektus. SY and SC were employed by the company Pluxity Co., Ltd.
